# Supplementary material for: Fetal influence on the human brain through the lifespan
Source: eLife. 2024 Apr 11;12:RP86812. doi: 10.7554/eLife.86812 (PMC11008813; doi:10.7554/eLife.86812)
Supplement: Supplementary file 1. [file elife-86812-supp1.docx]

| **Cortical measure** | **Compared Datasets** | **Birth Weight** | | | **Birth weight,**  **ICV-corrected** | | **Birth Weight,**  **(-log10(p))** | | | **Birth Weight x Time** | | |
| --- | --- | --- | --- | --- | --- | --- | --- | --- | --- | --- | --- | --- |
|  |  | **β** | | **p** | **β** | **p** | **β** | **p** | | **β** | | **p** |
| **Area** | LCBC - UKB | .51 | | <.001 | .24 | .010 | .35 | <.001 | | -.20 | | .073 |
|  | LCBC - ABCD | | .71 | <.001 | .61 | <.001 | .60<.001 | | -.05 | .770 |  |  |
|  | UKB - ABCD | .63 | | <.001 | .47 | <.001 | .54 | <.001 | | -.35 | | <.001 |
| **Thick-ness** | LCBC - UKB | .24 | | .011 | .25 | .006 | .23 | .005 | | -.04 | | .800 |
|  | LCBC -ABCD | .45 | | <.001 | .45 | <.001 | .43 | <.001 | | -.11 | | .430 |
|  | UKB-ABCD | .44 | | <.001 | .44 | <.001 | .37 | <.001 | | -.20 | | .150 |
| **Volume** | LCBC-UKB | .64 | | <.001 | .38 | <.001 | .45 | <.001 | | .08 | | .520 |
|  | LCBC-ABCD | .79 | | <.001 | .70 | <.001 | .66 | <.001 | | -.34 | | .005 |
|  | UKB-ABCD | .73 | | <.001 | .56 | <.001 | .60 | <.001 | | .03 | | .760 |

**Supplementary Table.** Spatial correlation of birth weight effects on brain structure across datasets. Pearson’s correlation and significance for pairwise spatial correlations between the different cohorts’ birth weight, with and without correction for ICV, and birth weight x time (years) -related cortical maps; i.e. effects of birth weight on cortical structure and change in cortical structure. Significance was assessed using the spin test. Spatial correlations were assessed using the Beta maps (and -log10(p) for birth weight). See Figure 3 and Supplementary Figure 9 and 10 for a visual representation. P-values are FDR-corrected (n = 9).
