# Supplementary material for: Fetal influence on the human brain through the lifespan
Source: eLife. 2024 Apr 11;12:RP86812. doi: 10.7554/eLife.86812 (PMC11008813; doi:10.7554/eLife.86812)
Supplement: Supplementary file 2. [file elife-86812-supp2.docx]

| **Measure** | **Datasets** | **Exploratory replicability** | **Confirmatory replicability** |
| --- | --- | --- | --- |
| Area | LCBC | .00 (.00) | .02 (.05) |
|  | ABCD | .00 (.00) | .68 (.35) |
|  | UKB | .00 (.00) | .00 (00) |
| Thickness | LCBC | .01 (.05) | .08 (.14) |
|  | ABCD | .01 (.14) | .56 (.25) |
|  | UKB | .00 (.01) | .00 (.02) |
| Volume | LCBC | .02 (.09) | .37 (.26) |
|  | ABCD | .00 (.02) | .60 (.27) |
|  | UKB | .01 (.03) | .01 (.03) |

**Supplementary Table.** Exploratory and confirmatory replicability of birth weight on cortical change within datasets. Units represent median and interquartile range.
