# Supplementary material for: Fetal influence on the human brain through the lifespan
Source: eLife. 2024 Apr 11;12:RP86812. doi: 10.7554/eLife.86812 (PMC11008813; doi:10.7554/eLife.86812)
Supplement: Supplementary file 3. [file elife-86812-supp3.docx]

| **Measure** | **Datasets** | **Type** | **Birth Weight_a 🡪 b_** | **Birth Weight_b 🡪 a_** | **BW x time_a 🡪 b_** | **BW x time_b🡪 a_** |
| --- | --- | --- | --- | --- | --- | --- |
| Area | LCBC - UKB | Exploratory | .94 | .86 | 0 | -- |
|  | LCBC – ABCD | Exploratory | .94 | .90 | 0 | 0 |
|  | UKB – ABCD | Exploratory | .91 | .94 | 0 | 0 |
| Thickness | LCBC - UKB | Exploratory | 0 | -- | 0 | 0 |
|  | LCBC – ABCD | Exploratory | .22 | .35 | 0 | 0 |
|  | UKB – ABCD | Exploratory | -- | 0 | 0 | 0 |
| Volume | LCBC - UKB | Exploratory | .85 | .76 | .002 | .012 |
|  | LCBC – ABCD | Exploratory | .93 | .81 | 0 | 0 |
|  | UKB – ABCD | Exploratory | .89 | .86 | -- | 0 |
| Area | LCBC - UKB | Confirmatory | .98 | .92 | 0 | -- |
|  | LCBC – ABCD | Confirmatory | .97 | .95 | .01 | .12 |
|  | UKB – ABCD | Confirmatory | .94 | .98 | -- | 0 |
| Thickness | LCBC - UKB | Confirmatory | .04 | -- | .27 | 0 |
|  | LCBC – ABCD | Confirmatory | .45 | .48 | 0 | .07 |
|  | UKB – ABCD | Confirmatory | -- | .09 | 0 | .002 |
| Volume | LCBC - UKB | Confirmatory | .94 | .87 | .15 | .22 |
|  | LCBC – ABCD | Confirmatory | .97 | .91 | 0 | .01 |
|  | UKB – ABCD | Confirmatory | .94 | .95 | 0 | .001 |

**Supplementary Table.** Exploratory and confirmatory replicability across datasets. “--" denotes no significant clusters in the right-hand dataset. _a 🡪 b_ and _b 🡪 a_ denotes directionality of the replicability analyses being “a” and “b” in the left and right hand of the “Compared Datasets” column. BW = Birth weight.
